# An Atypical Case of Hemolytic Uremic Syndrome Caused by Shiga Toxin Produced by Aeromonas spp.

**DOI:** 10.7759/cureus.61859

**Published:** 2024-06-06

**Authors:** Soloman R Kreik

**Affiliations:** 1 Nephrology, University of Michigan, Ann Arbor, USA

**Keywords:** general infectious diseases, general nephrology, nephrology, aeromonas hydrophilia, acute kidney injury, hemolytic uremic syndrome

## Abstract

Hemolytic uremic syndrome (HUS), traditionally recognized in pediatric populations, is characterized by renal insufficiency, hemolytic anemia, and thrombocytopenia, often linked to Shiga-like toxin (SLT) exposure. While typically associated with enteric pathogens like *Escherichia coli* (*E. coli*) and *Shigella*, *Aeromonas spp.* has also been identified as potential SLT producers, posing a new challenge. This study presents an exceptional case of HUS in a 77-year-old female, implicating *Aeromonas hydrophila* as the causative agent. The patient's clinical trajectory, marked by acute kidney injury post-consumption of raw oysters, underscores the unorthodox manifestation of HUS in adults. Diagnostic confirmation via stool antigen testing and blood culture revealed the presence of SLT and *Aeromonas hydrophila*, respectively. This case underscores the evolving landscape of HUS etiology, stressing the importance of heightened clinical awareness to expedite therapeutic intervention and mitigate long-term renal complications.

## Introduction

This article was previously presented as a poster presentation at the American College of Chest Physicians Annual Chest Meeting on October 9, 2023. Hemolytic uremic syndrome (HUS) is a leading cause of acute kidney injury in infants and young children, rarely affecting adults. It is defined as a triad of renal insufficiency, hemolytic anemia, and thrombocytopenia associated with a Shiga-like toxin (SLT). SLT is an antigen associated with diarrhea and plays a role in the pathogenesis of HUS. Typically, the toxin is generated by *Escherichia coli* (*E. coli*) or *Shigella* species. However, recent research indicates that Aeromonas species, which were previously known to cause infections mainly in immunocompromised individuals, can also produce active SLT [1,2]. We present an atypical case of HUS caused by SLT-producing *Aeromonas* *hydrophila*.

## Case presentation

**Figure 1:**
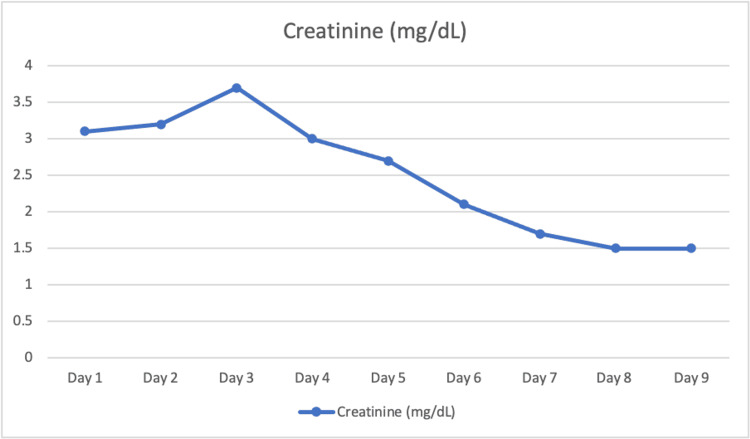
Creatinine trend

A 77-year-old female with a past medical history of hypertension presented with one day of worsening lethargy. The patient complained of bloody diarrhea and diffuse abdominal pain. She endorsed eating raw oysters a day prior. On examination, her abdomen was diffusely tender to palpation. Initial labs revealed a creatinine of 3.1 mg/dL, hemoglobin of 10.1 g/dL, and a platelet count of 102,000/µL. Complete blood count (CBC) and comprehensive metabolic panel (CMP) were otherwise unremarkable (Table 1, Figure 1).

**Table 1:** Laboratory results on admission

Component	Normal Range	Patient Values	Units
CBC			
White Blood Cell Count (WBC)	4,500 - 11,000	12,300	cells/µL
Red Blood Cell Count (RBC)	Female: 4.2 - 5.4	3.8	10^6 cells/µL
Hemoglobin (Hgb)	Female: 12.1 - 15.1	10.1	g/dL
Hematocrit (Hct)	Female: 36.1 - 44.3	33.5	%
Platelet Count	150,000 - 450,000	102,000	platelets/µL
Mean Corpuscular Volume (MCV)	80 - 100	82	fL
Mean Corpuscular Hemoglobin (MCH)	27 - 31	29	pg/cell
Mean Corpuscular Hemoglobin Concentration (MCHC)	32 - 36	32	g/dL
Red Cell Distribution Width (RDW)	11.5 - 14.5	16.1	%
CMP			
Glucose	70 - 100	135	mg/dL
Sodium (Na)	135 - 145	133	mmol/L
Potassium (K)	3.5 - 5.1	4.1	mmol/L
Chloride (Cl)	98 - 107	102	mmol/L
Carbon Dioxide (CO_2_)	23 - 29	25	mmol/L
Calcium (Ca)	8.5 - 10.5	9.1	mg/dL
Total Protein	6.0 - 8.3	7.1	g/dL
Albumin	3.4 - 5.4	4.2	g/dL
Total Bilirubin	0.1 - 1.2	1.4	mg/dL
Alkaline Phosphatase (ALP)	44 - 147	162	IU/L
Aspartate Aminotransferase (AST)	10 - 40	27	IU/L
Alanine Aminotransferase (ALT)	7 - 56	32	IU/L
Blood Urea Nitrogen (BUN)	7 - 20	23	mg/dL
Creatinine	0.6 - 1.1	3.1	mg/dL
Lactate dehydrogenase (LDH)	140 – 280	346	U/L
Haptoglobin	41 - 165	28	mg/dL

**Figure 2:**
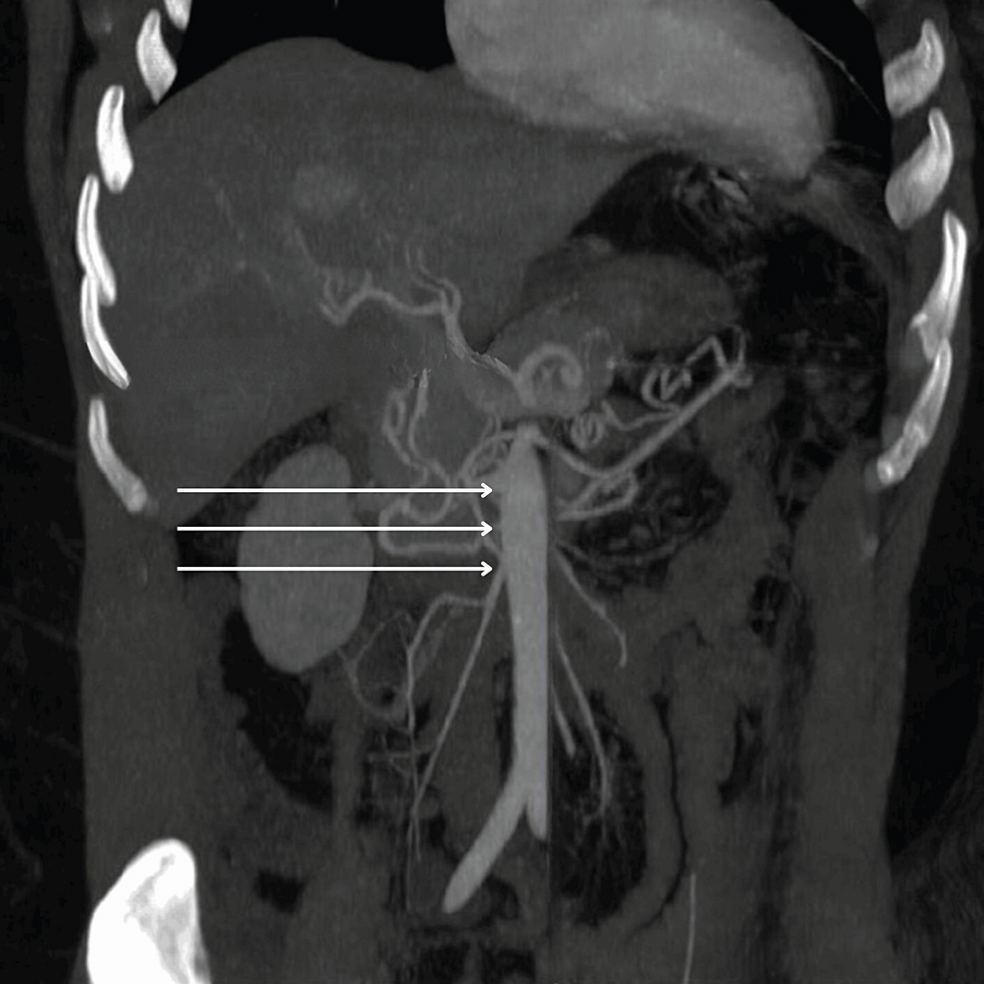
CT angiogram of the abdomen The image is depicting patent celiac, superior mesenteric, and inferior mesenteric arteries.

A CT angiogram of the abdomen and pelvis showed patent celiac, superior mesenteric, and inferior mesenteric arteries (Figure 2). The patient was bolused with normal saline and started on vancomycin and flagyl after blood cultures were drawn. Peripheral blood smear returned with schistocytes, lactate dehydrogenase (LDH) was elevated at 346 U/L and haptoglobin was low at 28 mg/dL. The patient’s renal status worsened until day 3, at which time the stool antigens returned positive for Shiga toxin and the blood culture showed *Aeromonas hydrophila* bacteremia. At day 9 the patient’s renal function subsequently returned to baseline, and she was discharged.

## Discussion

HUS is a microvascular occlusive disease that damages the glomeruli due to the accumulation of platelets and sheared red blood cells [1]. Isolation of *Aeromonas hydrophila-*produced SLTs is concerning, as these toxins are associated with HUS. *Aeromonas spp.* producing SLTs could be an emergent pathogen in the pathophysiology of HUS [2]. This was a rare phenomenon, which is now proven by PCR amplification of the *stx1* and *stx2* genes in many similar cases [3]. This case highlights the challenges in diagnosing atypical HUS. Our patient’s HUS diagnosis demonstrated a prodromal illness of diarrhea, thrombocytopenia, anemia, and development of renal failure. The pathophysiology of renal failure revolves around a cascade triggered by a direct insult of both SLT and the rupture of red blood cells. This process is believed to involve the toxin's journey through the bloodstream, targeting endothelial cells within the glomerular capillaries, mesangial cells, and tubular cells. The initial damage to endothelial cells sets off a series of events, including an increase in platelet aggregation and adhesion, ultimately leading to the formation of micro-thrombi [4]. This prothrombotic state exacerbates the injury, contributing to the progression of renal damage. Roughly two-thirds of individuals diagnosed with HUS need renal replacement therapy, and fortunately, most of them eventually recover renal function [5]. Conversely, around one-third of patients experience less severe renal complications and may not require dialysis treatment [6]. Early identification of HUS will allow prompt management and the prevention of long-standing renal failure.

## Conclusions

In summary, our case report emphasizes the importance of identifying *Aeromonas hydrophila* as a potential contributor to hemolytic uremic syndrome (HUS). By delving into the underlying mechanisms of HUS, we've highlighted the significance of microvascular occlusion and the production of Shiga-like toxins (SLTs) by *Aeromonas spp.* This underscores the shifting landscape of infectious agents associated with HUS, emphasizing the emergence of *Aeromonas spp.* as a noteworthy pathogen. Our findings serve as a crucial reminder of the dynamic nature of infectious diseases and the necessity of remaining vigilant in identifying novel causative agents.
